# Calcium Phosphate Nanostructured Biocomposites with Applications in Bone Tissue Engineering

**DOI:** 10.3390/ma19071375

**Published:** 2026-03-30

**Authors:** Gabriela Petcu, Elena Maria Anghel, Viorica Parvulescu, Alina Maria Holban, Carmen Curutiu, Cornelia-Ioana Ilie, Lia-Mara Ditu

**Affiliations:** 1 Institute of Physical Chemistry—Ilie Murgulescu of the Romanian Academy, Spl. Independentei 202, 060021 Bucharest, Romania; gpetcu@icf.ro (G.P.); manghel@icf.ro (E.M.A.); vpirvulescu@icf.ro (V.P.); 2 Department of Botany and Microbiology, Faculty of Biology, University of Bucharest, Portocalilor 1-3, 060101 Bucharest, Romania; carmen.curutiu@bio.unibuc.ro (C.C.); lia-mara.ditu@bio.unibuc.ro (L.-M.D.); 3 MICROGEN Research Centre, Faculty of Biology, University of Bucharest, Portocalilor 1-3, 060101 Bucharest, Romania; 4 Department of Science and Engineering of Oxide Materials and Nanomaterials, Faculty of Chemical Engineering and Biotechnologies, National University of Science and Technology POLITEHNICA Bucharest, 011061 Bucharest, Romania; cornelia_ioana.ilie@upb.ro; 5 National Research Center for Micro and Nanomaterials, Faculty of Chemical Engineering and Biotechnologies, National University of Science and Technology POLITEHNICA Bucharest, 1-7 Gh. Polizu Street, 011061 Bucharest, Romania

**Keywords:** biocomposites, calcium phosphates, hydroxyapatite, functionalization, ions substitution, polymers, bioglass, bioactivity, biocompatibility, bone regeneration

## Abstract

Nanostructured calcium phosphate-based (CaP) biocomposites have proven to be ideal candidates for the creation of multifunctional systems with applications in biomedicine. This review presents a critical and integrative overview of recent advances in the synthesis of CaP nanocomposites with applications in bone tissue regeneration. An analysis of calcium phosphate-based nanocomposites is thus provided by correlating their composition, synthesis routes and biological properties, guiding the rational development of next-generation biomaterials for bone tissue engineering. The first section presents calcium phosphates, such as hydroxyapatite (HAp) or β-tricalcium phosphate (β-TCP), used in the preparation of nanocomposite materials. Next, the main biocomposite materials are analyzed as a result of the functionalization of calcium phosphates by metal ion substitutions or by the addition of polymers, bioglass or metal additives. Thus, biomaterials with excellent properties in applications such as tissue engineering have been obtained. The synergistic effect of materials in the composition of biocomposites favored the improvement of properties such as bioactivity, mechanical strength, antimicrobial activity, structure and porosity. Beyond classical osteoconductivity, CaP-based nanocomposites demonstrate a broad spectrum of biological activities like immunomodulatory effects, pro-healing signaling, anti-inflammatory pathways, antibacterial and antifungal mechanisms, and capabilities for precise drug delivery or theranostic applications.

## 1. Introduction

Tissue engineering and regenerative medicine are rapidly evolving fields of research, in which biomimetic scaffolds play a central role by providing three-dimensional templates that guide tissue regeneration [1,2,3]. Biomaterials used as scaffolds are generally classified into two main categories: natural biomimetic materials [3] and synthetic [4,5,6]. Natural polymers, such as collagen, alginate, or chitosan, exhibit excellent bioactivity, but often suffer from low mechanical strength. In contrast, synthetic biomimetic materials are designed to replicate the hierarchical architecture and biological signals of the extracellular matrix (ECM), combining mechanical robustness with tunable biofunctionality. They are obtained using synthetic polymers that incorporate bioactive factors (peptides, growth factors), achieving controlled cellular interaction and improved functionality. The development of a bone implant material with chemical stability and antibacterial resistance is a particular requirement. To date, multiple studies have been conducted on the obtaining and use of different materials for bone regeneration, but improving tissue integration and accelerating the healing process is still a challenge. In most cases, bacterial infection is a determining factor for delaying healing processes. Thus, a main objective pursued was to overcome the limits of traditional implants, such as immune rejection, and the results obtained are significant. An important role in this regard was represented by the imitation of the natural nanoscale architecture of bone (collagen/apatite composites), which is inherently nanostructured. Biomimetic materials with compositions or properties similar to those of the ECM, new technologies for their processing, strategies for their biological administration, and wound healing programs have been developed by imitating the ECM, and natural cell adhesion was promoted, building new tissues [4,5,6]. Thus, the functionalization of materials was achieved to generate bioactivity by incorporating bioactive molecules (peptides, growth factors), which allowed the creation of living implants. Natural materials such as collagen, silk, chitosan, or hyaluronic acid have also been used to form scaffolds that naturally resemble tissue. Calcium phosphates (such as HAp, β-TCP) are biomaterials with bone-like composition, high bioactivity, osteoconductivity, and biocompatibility. However, they face limitations such as poor mechanical strength (fragility) for load-bearing applications, slow degradation in dense forms, and complex cellular interactions that require surface modification for optimal integration and inflammation control. This is why research has focused on composites, nanostructuring, and surface functionalization to obtain stronger materials and improve their bone integration and regeneration [7,8]. The transition from classical materials to nanostructures and its impact on mechanical properties, cell-material interactions, dissolution, and reprecipitation kinetics represents a particular challenge for the development of new materials with properties close to the natural bone structure. Thus, the use of nanostructured calcium phosphates in tissue engineering has significantly improved bone regeneration by mimicking the nanoarchitecture of natural bone [9]. This has resulted in superior mechanical properties, improved cellular interaction, and improved biointegration. Nanostructuring enhances bioactivity by increasing the surface-to-volume ratio and introduces unique properties not observed in bulk composite materials. This opens up more opportunities for better protein adsorption and enhanced cellular responses, leading to enhanced implant performance [10,11]. Better protein adsorption and enhanced cellular responses lead to enhanced implant performance. Nanoparticles (NPs) can be easily modified with drugs, proteins, or magnetic ions, enabling targeted delivery and advanced therapies. Nanostructures also promote better cellular integration and reduced inflammation compared to macromaterials. They allow for precise control over surface energy, wettability, and ion/protein interaction. Unfortunately, NPs tend to agglomerate, requiring specific processing to maintain nanoscale characteristics. Functionalization and doping of CaP composites with ions enhance their properties for applications such as bone tissue engineering [12]. Doping with metal ions, such as Mg^2+^, Sr^2+^, and Zn^2+^, can enhance mechanical strength and promote bone growth, while other ions, such as Ag^+^, Cu^2+^, and Zn^2+^, can promote antibacterial effects [13,14,15,16]. In addition, functionalization may involve other strategies, such as the incorporation of bioactive molecules or coatings to tailor performance further [17,18,19,20]. Multifunctionality in the context of CaP composites results in properties that mimic biological materials, and the surface chemistry can be tailored for diverse biomedical applications [18,21,22,23]. These multiple functions are driven by the nanostructured composite materials (CaP + polymers, metals, oxides, carbon-based materials). These multiple functions are driven by the nanostructured composite materials (CaP + polymers, metals, oxides, carbon-based materials. Although there are a large number of publications on these materials (Table 1), an integrated perspective that correlates their characteristics in terms of structure, properties, and functionality is lacking. Also, a gap in the existing literature on these materials consists of a limited number of comparisons between different types of composites and their multiple functions.

This review aims to provide an integrated overview of calcium phosphate nanocomposites by correlating their composition, synthesis routes, structural features, and multifunctional properties with their biomedical performance. Therefore, this review provides a unified framework for calcium phosphate nanocomposites by correlating their composition, synthesis routes, structural features, and biological properties, thereby guiding the rational development of next-generation biomaterials for bone tissue engineering.

## 2. CaPs Base Matrix of the Biomedical Nanocomposites

Calcium phosphates (CaPs) have been widely studied over recent decades for their biomedical potential, particularly in the treatment of bone- and dental-related disorders [33,34]. Their clinical application spans a broad spectrum of medical procedures, including the management of bone defects and fractures, total joint arthroplasty, spinal surgery, dental implant placement, periodontal therapy, and cranio-maxillofacial reconstruction [35]. CaPs-based biomaterials are available in a variety of chemical compositions and structural phases, such as dicalcium phosphate dihydrate (brushite, DCPD, CaHPO_4_·2H_2_O), TCP (Ca_3_(PO_4_)_2_), which exists in three crystalline polymorphs (α-TCP, α′-TCP, andβ-TCP), tetracalcium phosphate (hilgenstockite, TTCP, Ca_4_(PO_4_)_2_O), octacalcium phosphate (OCP, Ca_8_H_2_(PO_4_)_6_·5H_2_O), amorphous calcium phosphate (ACP), HAp (Ca_10_(PO_4_)_6_(OH)_2_), as well as various combinations thereof [34,36]. Table 2 summarizes the main types of calcium phosphates, highlighting their characteristic physicochemical properties and representative biomedical applications. Among the various calcium phosphate compounds, HAp and β-TCP have been the most extensively studied [35]. Calcium phosphate materials can be obtained via various chemical routes, including combustion, co-flocculation, and solid-state [36], sol–gel, co-precipitation, emulsion, hydrothermal, mechanochemical, ultrasonic, template-assisted, and microwave-assisted methods [33]. Green or bio-derived approaches have also been explored for obtaining HAp, TCP, DCPD, and monocalcium phosphate monohydrate, using waste resources such as bones, cockle shells, or eggshells [36,37]. Nevertheless, for biomedical applications demanding exceptionally high purity, chemically synthesized CaPs remain the preferred route over extraction-based methods [17].

**Figure 1:**
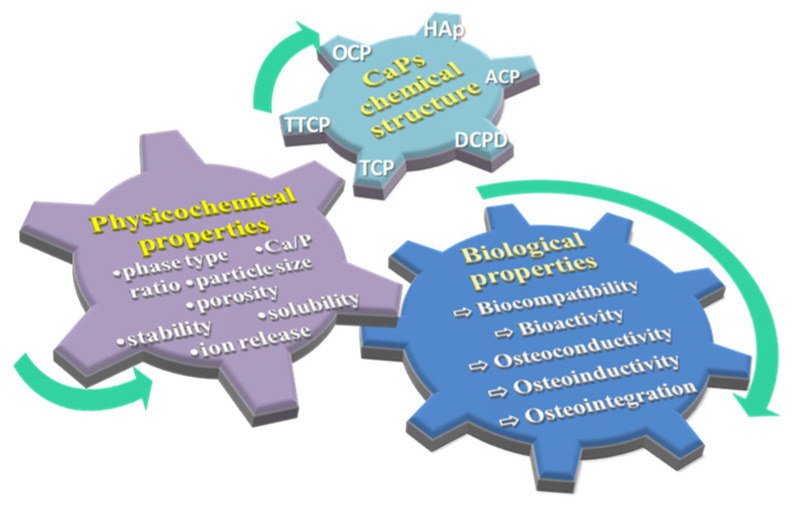
Interdependence of chemical structure, physicochemical characteristics, and biological performance in CaP.

The medical relevance of CaPs is primarily attributed to their chemical resemblance to the mineral phase of native bone and teeth, which supports their ability to facilitate bone regeneration. Due to their structural similarity to hard tissues, calcium phosphates exhibit osteoconductive properties and a pronounced affinity for the adsorption of proteins and growth factors [15,35]. Moreover, their excellent biocompatibility and bioactivity further underline their suitability for use in biomedical applications [15]. Each calcium phosphate phase exhibits distinct physicochemical characteristics that define its biological response. Variations within a given CaP, such as the different structural forms of OCP or the crystalline polymorphs of TCP, result in specific material fingerprints that directly influence biological behavior [34]. Notably, even chemically identical and structurally related phases, such as α- and β-TCP, display substantial differences in crystal structure, density, and solubility, leading to divergent biological performance and clinical relevance [39]. β-TCP, characterized by lower solubility and higher structural stability, is typically employed in the fabrication of dense or macroporous granules and blocks for bone repair and regeneration. Conversely, the greater solubility and reactivity of α-TCP make it particularly suited for use as a fine powder in calcium phosphate cements (a mixture of calcium phosphate compounds with a liquid phase for biomedical purposes [40]), although bulk α-TCP constructs are also commercially available. These distinctions underscore how the specific physicochemical traits of each TCP phase dictate their respective clinical applications [39]. The relationship between chemical structure, the main physicochemical characteristics, and biological performance of CaP materials is schematically suggested in Figure 1.

The biological performance of calcium phosphate materials depends not only on their chemical type, but also on concentration, particle size, micro- and nanostructure, phase composition, and manufacturing method [34,35]. All types of CaPs can be produced in various forms, including dense or porous bulk constructs, granules, powders, or coatings, each offering distinct surface characteristics and porosity that affect cellular responses and biocompatibility [35,44]. Smaller particle sizes and higher specific surface areas, typical of nanoscale materials, can increase material reactivity but may reduce cell viability if too extreme. Conversely, larger particles and more stable structures tend to support higher cell survival. Similarly, materials processed into cements or gels often show reduced cellular activity compared to powders, likely due to altered solubility and ion release [45]. The porosity and bioactive surface of CaP materials influence biological performance by enhancing cell adhesion, proliferation, and differentiation, which in turn improves biocompatibility, osteoconductivity, and, in some cases, osteoinductivity, ultimately promoting bone formation [38]. These observations highlight that both the intrinsic properties and the form of calcium phosphate critically influence its biological behavior [34].

Another important parameter that dictates the biomedical application of CaPs biomaterials is the Ca/P ratio, which strongly influences the properties of the calcium phosphate cements, such as the solubility and the reaction kinetics during setting. HAp, with a high Ca/P ratio (1.67), represents the final binding phase in many bone cements and provides excellent biocompatibility and long-term stability due to its chemical similarity to natural bone mineral [15,46,47,48,49]. TCP, with a Ca/P ratio of 1.5, offers a balance between structural stability and moderate resorption, allowing gradual conversion into more durable HAp under physiological conditions [50,51,52]. DCPD, with the lowest Ca/P ratio of 1.0, is employed in fast-setting cements, where its high solubility promotes the release of calcium and phosphate ions and enhances osteointegration [15,53]. In addition, reducing the particle size of the cement powder and increasing the powder-to-liquid (P/L) ratio have been shown to improve mechanical strength and accelerate the solidification of calcium phosphate cements [54].

DCPD, also known as brushite, is a calcium phosphate phase with significant biological relevance in bone and dental biomineralization. Both hydrated (brushite) and anhydrous (monetite) forms have been extensively studied due to their involvement in mineral metabolism of hard tissues. DCPD is characterized by a relatively low Ca/P ratio, which contributes to its high solubility and rapid degradability compared to other calcium phosphates. This pronounced resorption behavior promotes extensive bone remodeling in vivo, making DCPD particularly attractive for bone tissue engineering applications. Moreover, its dissolution and growth mechanisms are of special interest in dental and orthopedic regeneration, and the material has found practical use in dental cements and restorative formulations [38].

Tricalcium phosphate is a widely used calcium phosphate bioceramic that exists in several crystalline polymorphs, including α-TCP, α′-TCP, and β-TCP. Owing to its chemical similarity to the mineral component of bone, TCP exhibits excellent biocompatibility and osteointegration [40]. It is frequently applied as a bone graft substitute in orthopedic, dental, and reconstructive surgeries [55]. One of the key advantages of TCP is its relatively high resorbability under physiological conditions, which allows gradual material degradation accompanied by progressive bone ingrowth and site recolonization. This balance between biodegradation and new bone formation makes TCP a suitable temporary scaffold for bone regeneration [40]. Tetracalcium phosphate, also known by its mineral name hilgenstockite, is a calcium phosphate bioceramic primarily used in biomedical applications related to bone repair. TTCP is commonly incorporated into CaP–based bone cements, where it is combined with other CaP phases and a liquid component to form injectable or moldable materials. Its reactivity and alkaline nature make it suitable for cement formulations that harden in situ, enabling effective filling of bone defects and promoting local mineralization [36].

Octacalcium phosphate has been widely proposed as a key precursor phase in biological apatite formation, particularly during the transformation toward HAp from supersaturated calcium and phosphate solutions [41]. Synthetic OCP has been shown to possess pronounced osteoconductive properties and can act as a nucleation site for initial bone deposition when implanted in bone defects [56,57]. Additionally, OCP is considered a metastable phase under physiological pH conditions, which thermodynamically favors its gradual conversion into HAp [41]. Due to these characteristics, OCP is regarded as a highly promising bone substitute material, with superior bone formation potential compared to more stable phases such as HAp and β-TCP [58,59]. ACP is a non-crystalline polymorph of calcium phosphate that typically appears as a transient intermediate during CaP precipitation processes. ACP is recognized as a crucial precursor in bone formation, preceding the development of more crystalline apatite phases [42]. Despite its biological importance, the practical application of ACP in bone regeneration has been limited by its low stability. In aqueous environments, ACP readily transforms into HAp [41,42], with the transformation kinetics being strongly influenced by parameters such as pH, temperature, and the presence of ionic species or additives [42].

Hydroxyapatite is one of the most extensively used CaP bioceramics for bone and dental tissue reconstruction due to its excellent biocompatibility, bioactivity, and osteoconductivity [38]. Its chemical resemblance to the mineral phase of natural bone enables strong bonding with host tissue, enhancement of enzymatic activity (alkaline phosphatase, ALP), and stimulation of osteogenic signaling pathways, including bone morphogenetic protein (BMP) expression and stem cell differentiation via ion release. Variants such as calcium-deficient hydroxyapatite (CDHA) further improve osseointegration through tunable Ca/P ratios and surface ion-exchange capabilities [43]. However, crystalline HAp exhibits slow degradation kinetics, limited mechanical strength, high brittleness, and low fracture toughness, resulting in reduced bioresorption compared to other, more soluble calcium phosphate phases [38].

Although many CaP biomaterials have traditionally been developed as single-phase systems, biomaterials with improved biological and functional properties have been obtained through the combination of two CaP phases exhibiting different physicochemical behaviors. Such systems can be regarded as composite biomaterials, as they are formed by constituents with distinct properties [35], and are commonly referred to as biphasic calcium phosphates (BCPs). For example, by integrating HAp phase with favorable mechanical properties and similarity to bone mineral with β-TCP for its favorable biodegradation and chemical stability, the resulting BCP biomaterial offers clear advantages over monophasic CaP ceramics, including enhanced bioactivity, tunable biodegradability, improved osteoconductivity, and better synchronization between material resorption and new bone formation [35,60,61]. BCPs offer the flexibility to modify the proportion of stable versus biodegradable phases, enabling fine-tuning of degradation and enhancing bone regeneration in specific applications [35,61].

The optimal ratio established by experts in the field for achieving desirable properties in bone regeneration is β-TCP/HA 80/20 (wt%) [61]. Deviations from this balance can lead to suboptimal outcomes: higher β-TCP content may induce proinflammatory responses and impair cell adhesion, although it enhances alkaline phosphatase (ALP) expression, a key factor in early-stage bone cell differentiation. In contrast, lower β-TCP levels support sustained viable cell adhesion over a longer period [61]. The β-TCP/HA ratio in BCP can be tailored by adjusting the calcium-to-phosphate (Ca/P) ratio of the precursor materials. Theoretically, changes in the Ca/P ratio determine the resulting phase composition, with a value of 1.5 corresponding to 100% β-TCP and 1.67 to 100% HA. The relationships described by Equations (1) and (2) further indicate that the HA/β-TCP ratio can be systematically varied as a function of the Ca/P ratio [61,62].

% HA = 600 × Ca/P − 900(1)

% β-TCP = 1 − (600 × Ca/P − 900)(2)

Notable advantages are recognized for biphasic calcium phosphates (BCPs) compared to other CaP ceramics, owing to their controllable dissolution behavior, adjustable mechanical properties, and balanced resorption/solubilization, which ensure material stability while supporting new bone formation [38]. BCP can be synthesized using several approaches, including high-temperature sintering [35], of non-stoichiometric calcium phosphates such as amorphous calcium phosphate or CDHA, as well as solid-state reactions between two solid compounds at elevated temperatures [63,64,65]. Mechanical mixing of HAp and β-TCP powders or their precursors is another possible method [66,67,68], although it may lead to inhomogeneous phase distributions and less reproducible material structures [35].

Recent research has increasingly focused on the fabrication of nanoscale CaP materials, driven by their ability to closely emulate the hierarchical composition of natural bone, which consists of an organic collagen matrix and inorganic CaP nanocrystals [35]. Reducing CaP to the nanoscale offers significant advantages, including enhanced bioactivity, osteoblast adhesion, proliferation, differentiation, and improved osseointegration, as well as more efficient deposition of calcium-containing minerals on the material surface [69,70]. These properties contrast with conventional microscale ceramics, which often exhibit lower biological performance and altered biodegradability and mechanical behavior. Furthermore, nanoscale CaPs serve as the base matrix of multifunctional composites, enabling the integration of organic or inorganic constituents to fine-tune mechanical strength, biodegradability, and cellular responses, thereby broadening their potential clinical applications in bone repair, regeneration, and tissue engineering [71]. Therefore, calcium phosphate-based nanobiomaterials with biomimetic structures and functionalities are expected to exhibit high bioactivity and favorable biological effects, ultimately serving as effective biomedical devices for bone repair and regeneration [33].

## 3. Nanocomposites Obtained by CaP Materials Functionalization

**Figure 2:**
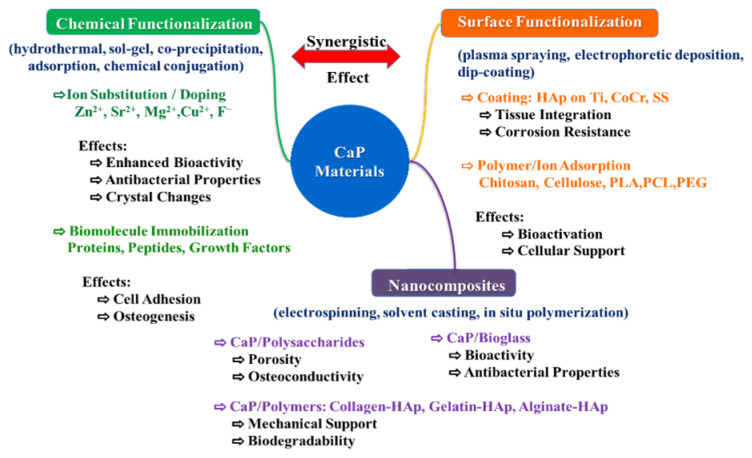
CaP functionalization strategies.

Functionalization of CaP materials involves modifying their surface or bulk composition with organic molecules or ions to enhance biocompatibility and bioactivity, particularly for orthopedic and dental implants. Techniques include surface coating, ion substitution, and biomolecule immobilization to improve cell adhesion and bone regeneration. Surface functionalization of CaP materials is often necessary to ensure more efficient integration with biological tissues and enhance the role of immobilized biomolecules in modulating immune responses [72]. Figure 2 provides a schematic overview of the main CaP functionalization strategies. Ion substitution is performed within the CaP lattice during synthesis, while surface coating and adsorption techniques modify only the surface of the CaP material to enhance biological interactions. In addition, biomolecule immobilization is achieved through chemical bonding of proteins and peptides onto the surface, whereas nanocomposite approaches involve the integration of CaP with polymers or other bioactive phases to improve both mechanical and biological performance. Composite materials have been developed using several methods of functionalizing CaPs, such as ion substitution, coating, immobilization of biomolecules, and combination with polymers. A large number of studies have investigated the effects of replacing Ca^2+^ or PO_4_^3−^ ions in the CaP network with bioactive ions such as Zn^2+^ and Cu^2+^ to provide antibacterial properties and enhance osteogenesis. Many of these materials have been used as functional coatings on metal substrates, thus resulting in different composites. Another type of composites has been developed either by combining CaP with natural or synthetic polymers, or by attaching growth factors such as proteins or peptide sequences, to improve osteogenesis and cell attachment. In this section, such nanocomposite biomaterials with applications in bone tissue engineering will be presented.

Research into high-quality implant materials has highlighted the important role of metallic implants. Metals and their alloys are crucial for orthopedic and dental implants due to their high mechanical strength, corrosion resistance, and biocompatibility. Titanium and its alloys (Ti-6Al-4V) are among the most widely used due to their excellent corrosion resistance, strength-to-weight ratio, and biocompatibility [73]. Cobalt-chromium (CoCr) alloys are often used for artificial joints (hip, knee) due to their superior wear and corrosion resistance. Stainless steel (316L) is a common and cost-effective option for temporary orthopedic fixation devices such as plates, screws, and pins [9]. Noble metals (gold and silver) are used in dentistry. Among shape memory alloys, nickel-titanium (NiTi) alloys are used in orthodontic wires and vascular stents. For sufficient resistance to corrosive environments and bioactivity, these metal components are coated with ceramic materials such as HAp, which is one of the main constituents of natural bone, having excellent biocompatibility and bioactivity [74,75]. In addition, synthetic HAp is very tolerant to ionic substitutions of divalent and trivalent cations in its network. This results in materials that closely resemble natural bones or teeth, with improved properties. The functional performance of calcium phosphate–based biomaterials is strongly influenced by their chemical versatility. This compositional adjustment enables nanoscale calcium phosphates to better emulate natural bone mineral and to elicit targeted biological responses. Consequently, ion substitution and doping are increasingly recognized as essential design parameters for CaP NPs, and their effects on material properties and biological activity are examined in this section.

### 3.1. Ion Substitution of the CaPs Matrix

Modification of the crystal structure through ion substitution or doping represents a key strategy for more accurately reproducing the characteristics of biological apatite [76]. Although HAp is chemically similar to the mineral component of bone, it differs from native apatite, which is nanocrystalline, non-stoichiometric, and contains a wide variety of substituted ions. The introduction of different cations or anions into the HAp lattice alters fundamental material properties, including crystallinity, lattice parameters, crystal size, surface charge, stability, and solubility, thereby influencing interfacial processes and biological interactions [76,77].

**Figure 3:**
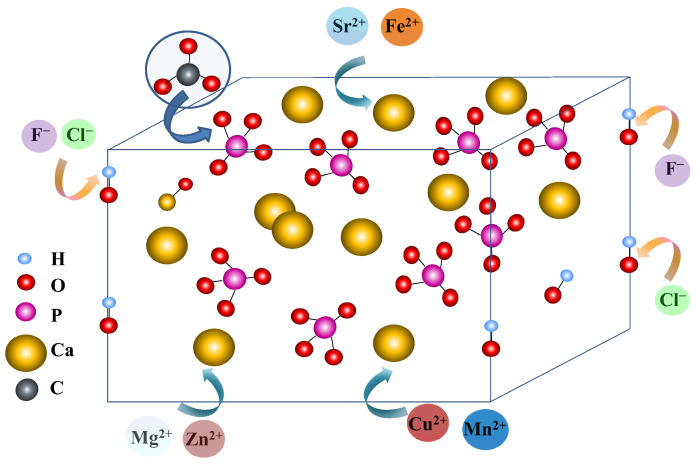
Representation of the ions’ substitution in the CaP matrix.

Ion-substituted HAp represents a promising approach for future clinical applications, as these materials can be incorporated into various dental products for therapeutic purposes [78,79,80]. The inorganic components of teeth contain essential trace elements such as magnesium, iron, zinc, sodium, silicate, and carbonate. Ion exchange can be performed with cations such as Sr^2+^, Mg^2+^, Fe^2+^, or Pb^2+^, which share the same oxidation state as Ca^2+^, or with anions such as F^−^ or Cl^−^, which possess the same charge as the OH^−^ group they replace (Figure 3). Ions may be incorporated into the HAp lattice through various chemical and physical mechanisms. This process is influenced by the charge and ionic radius of the substituting ions. For example, substitution of Ca^2+^ with Sr^2+^ is favored due to their similar ionic radii (1.18 Å for Sr^2+^ and 1.00 Å for Ca^2+^), whereas substitution with Mg^2+^ is less favorable because of its smaller ionic radius (0.72 Å). In cases involving ions with different charges, such as the substitution of PO_4_^3−^ with CO_3_^2−^, vacancies are created, leading to the simultaneous release of Ca^2+^ and OH^−^ ions [79,80,81,82,83,84]. Most ions employed in substitution processes are cations (Table 3). Table 3 summarizes various calcium phosphate–based materials substituted using different synthesis methods. In addition to their physicochemical properties, numerous studies have investigated the effects of ionic substitution on application-relevant properties, particularly in the field of bone tissue engineering.

The synthesis method and associated processing conditions used for HAp preparation significantly influence ion incorporation [85,86,87]. In the co-precipitation method, where calcium and phosphate are simultaneously precipitated in the presence of the substituent ion, a more homogeneous distribution of the dopant is typically achieved. In contrast, when the substituent ion is introduced during HAp formation via high-temperature sintering, a less homogeneous structure is generally obtained. It has also been demonstrated that not only the nature of the substituted ions but also their concentration significantly affects the properties of HAp. For example, in HAp coatings deposited on TiO_2_/Ti substrates, notable topographical modifications occur following ionic substitution [88,89,90,91]. Whereas unsubstituted HAp consists of flake-like particles with micrometric dimensions, the substitution of calcium ions with strontium ions results in the formation of spherical particles. Additionally, biomineralization processes enable the incorporation of strontium, silicon, and fluoride ions into apatite coatings, leading to the development of distinct morphologies, including spherical, acicular, and nanoflake structures. Ion substitution also affects crystal size, with biomimetic HAp coatings generally exhibiting reduced crystallite dimensions after doping. Partial substitution of OH^−^ ions with F^−^ ions results in an acicular morphology closely resembling that of dental enamel.

**Table 3:** Examples of the ionic substituted CaP materials and their properties with medical applications.

Substituted CaP	Preparation Method	Properties	Applications	Ref.
La/Cu substituted HAp coating on Ti	electrodeposition	higher antimicrobial and biological	orthopedic fields	[77]
La -doped HAp	sol–gel	higher electrical and ferroelectrical	coating of metallic implants	[78]
Sr substituted CaP	gelatin	variation in the osteoclast activity and osteoblast selectively	surgical treatment and care for large bone defects	[79]
Mg^2+^, Mn^2+^/CaP	wet precipitation	biological, not affected osteoblastic cells	suitability for bone tissue engineering applications	[15]
Ag^+^/CaP	impregnation	antibacterial properties	stabilization of implants	[16]
Zn^2+^, Mg^2+^/HAp	wet precipitation	non-cytotoxic, unique biological; antibacterial properties	implants for tissue regeneration	[13]
xZn^2+^/HAp x = 0.2, 0.6, 1.0, 1.5, 2.0	mechanochemical synthesis	high biocompatibility, lowest cytotoxicity	bone tissue regeneration	[82]
Sr^2+^, Mg^2+^, Zn^2+^/HAp	biomineralization	biocompatibility	coating titanium-based implants	[91]
Sr, Si and F/HAp	biomineralization	bioactivity, biocompatibility	coating metallic implants	[88]
La^3+^, Sm^3+^, Gd^3+^, Ho^3+^, Yb^3+^ and Lu^3+^/HAp	isothermal titration calorimetry ion exchange	bioactivity, biocompatibility	Coating metallic implants	[89]
Mg^2+^, Sr^2+^ and (CO_3_)^2−^/HAp	wet-precipitation	amphoteric surface	Biomedical implants	[92]
Cu^2+^/HAp coated on Ti nanotube	electrochemical	antibacterial properties	antibacterial and osteogenic applications	[93]
Ag^+^/HAp; Fe^2+^/HAp	sol–gel	bioactive behavior	tissue engineering applications	[94]
Mg^2+^, Cu^2+^/HAp/CFR-PEEK *	sol–gel	antibacterial properties	biomedical implants	[95]
Sr-HAp	wet chemical precipitation	bioactivity	biomedical applications	[96]

* carbon fiber reinforced polyetheretherketone.

In the case of wet chemical synthesis, it was observed that the substitution of calcium with strontium ions led to an increase in the unit cell parameters of the HAp and its cell volume, showing a linear dependence with increasing Sr concentration. On the other hand, a lower amount of strontium (10 and 20% mol) caused a decrease in the crystallite domain size compared to the initial HAp, while at a higher Sr content (40% mol), a significant increase in the crystallite domain size was observed. Compared to these results, Si-substituted HAp showed a morphology similar to pure HAp, but the particle size decreased from micrometric to nanometric scale [97]. Other studies, however, reported preservation of morphology for a strontium-doped apatite layer [91]. It was found that the concentration of strontium ions also affects the morphology of the coatings. Lower Sr concentration resulted in irregular and porous spherical particles, while higher concentration resulted in regular spherical particles. In addition, after ion substitution, the biomimetic HAp coatings exhibited poor crystallinity, and the crystal size decreased. In conclusion, the surface composition and morphology of HAp obtained by biomineralization can be controlled using ion substitutions. It was also observed that the association of Ca^2+^ cations and OH^−^ vacancies forms Lewis acidic sites, while the P–OH cations on the HPO_4_^2−^ and H_2_PO_4_^−^ surface form Brønsted acidic sites [92]. The study of these systems showed that the incorporation of foreign ions Mg^2+^ and Sr^2+^ into the HAp structure can have an impact on the amphoteric nature of the surface. Similar to Ca^2+^ cations, Mg^2+^ and Sr^2+^ ions can act as Lewis acidic sites whose acidity varies depending on the ionic radius: Mg^2+^ > Ca^2+^ > Sr^2+^. It was also demonstrated that carbonate ions modified the amphoteric nature of HAp by increasing the number of basic sites, while reducing the acidic sites.

The previous studies evidenced the different role of substituted ions due to their effects on the HAp structure, which influences its properties. Thus, both Sr^2+^ and Mg^2+^ can modify the network parameters and crystallinity of HAp, thus influencing its solubility, bioactivity, and biodegradability [86]. The presence of Zn^2+^ and Si^4+^ ions, however, can increase the antibacterial properties of the material, as well as enhance its ability to bind to bone. Thus, zinc plays an important role in tissue growth and development and is involved in various processes, such as cell division or gene transcription. Consequently, its deficiency can lead to a compromised immune system and serious pathologies. Hydroxyapatite network containing carbonate, Fe^2+,3+^, Sr^2+^, Mg^2+^, or Si^2+^ ions can be used to improve bone healing, while the release of Sr^2+^, Ag^+^, Zn^2+^, Co^2+^, and Cu^2+^ ions from the HAp network has demonstrated antibacterial activity [93,94,96]. The introduction of Er/Ce and Sr/Ni into the HAp network had a significant effect on the biocompatibility, while the introduction of Sr/Sm and Eu influenced the mechanical properties of HAp [96].

Since, in many cases, implant failure is due to the deposition of antibiotic-resistant bacteria, there has been a rapid development of strategies to inhibit early bacterial infection on implants in the orthopedic field. Thus, in the case of titanium implants, antibacterial properties have been improved by coating with pure HAp or substituted ions [94]. Moreover, copper (Cu)-based NPs have usually been considered a competitive candidate for the modification of Ti-based implants due to their simple synthesis process and broad-spectrum antibacterial capacity. Thus, Cu-substituted HAp NPs have been prepared and then deposited on titanium dioxide nanotube substrates. They have demonstrated antibacterial properties against *Escherichia coli* (*E. coli*) and *Staphylococcus aureus* (*S. aureus*) [93].

The improvement of osteogenic and antibacterial properties was achieved in the case of carbon fiber reinforced polyetheretherketone (CFR-PEEK) implants by coatings with bovine bone-derived HAp co-substituted with Mg^2+^ and Cu^2+^ [95]. The surface characterization and osteogenic and antibacterial properties of SCP-BHAMgCu samples were investigated in vitro. In this case, antibacterial properties were also proven, especially for *E. coli* and *S. aureus*. Composite materials with improved osteogenic and antibacterial properties were thus obtained. These results highlighted the possibility of sustainable use of bovine bone waste, but also highlighted the role of functional coatings for biomedical implants.

### 3.2. Nanocomposite Materials Containing CaPs

Nanocomposites based on ceramic NPs, such as CaPs, are essential for their applications in regenerative medicine [28,29,30,31,32]. This is because the use of scaffold materials of natural origin, such as polysaccharides (chitosan or hyaluronic acid), is often compromised due to their low mechanical and chemical stability [26]. On the other hand, synthetic materials, such as polyethylene glycols, are bioinert and thus cannot support cell adhesion and tissue development. Therefore, their bioactivation is necessary. In both cases, the addition of ceramic NPs has proven useful [98]. By nucleating these inorganic phases on organic biomolecules, various hybrid materials have recently been obtained. Since the speed of regeneration is important in the recovery of artificial implants or in the healing of bone fractures, materials with an activating role have been obtained. Inorganic/organic hybrid materials containing CaPs have been noted in this regard [98]. These materials are obtained by functionalizing CaP NPs. Functionalization involves modifying the surface chemistry of CaP materials to attach specific molecules or control surface properties, often by covalent or adsorption methods. The former involves the creation of stable covalent bonds between the CaP surface (silica-coated or not) and a functional molecule (proteins or polymers). Adsorption is a simpler method, in which molecules are adsorbed onto the surface of the CaP material. This is commonly used to functionalize CaP NPs with specific polymers or ions for applications such as remineralization in dentistry. Functionalization can confer various properties to CaP materials, including enhanced osteogenic or antimicrobial activity. CaP-based nanocomposite biomaterials have developed as a versatile platform for both dental and orthopedic applications, offering the possibility of combining the bioactivity of inorganic phases with the functional properties of organic polymers. ACP, for example, has been successfully incorporated into nanocomposite coatings and dental resins, taking advantage of its ability to release calcium and phosphate ions in situ, which promotes enamel remineralization and supports adhesion [99]. A synergistic effect was evidenced between doping of CaPs and their functionalization. This effect significantly improves biological, structural, and chemical properties, such as enhanced bone regeneration, personalized drug delivery, and increased surface reactivity. Doping introduces ions to modify the network, while functionalization adds surface molecules to enhance bioactivity, providing a combined effect superior to either method used individually.

CaP materials have been combined with polymeric materials, such as collagen, polyamide, poly(lactic acid), and poly(lactide-co-glycolide) to produce nanocomposites [3,18,100,101,102]. Natural biodegradable polymers have also been used because they have better biocompatibility than synthetic polymers. Synthetic hydrogels used in tissue engineering can be formed from a range of hydrophilic synthetic polymers or polysaccharides, including poly(ethylene glycol) (PEG), poly(vinyl alcohol) (PVA), poly(hydroxyethyl methacrylate) (poly(HEMA)), and alginate derivatives.

#### 3.2.1. CaPs/Polysaccharide Biocomposites

Chitosan is a natural polysaccharide that has attracted attention due to its abundant sources and bioactivity [2,4]. It is a leading biomaterial for tissue engineering scaffolds due to its biocompatibility, biodegradability, non-toxicity, and antibacterial properties. Chitosan promotes cell adhesion, proliferation, and differentiation. At the same time, its porous structure promotes the repair of bone, skin, cartilage, and nerve tissues. However, pure chitosan materials exhibit insufficient mechanical properties, which affects the stability and integrity in vivo when used as scaffolds for tissue engineering. Although of interest in applications, pure chitosan scaffolds can often exhibit low mechanical strength. Combining chitosan with a ceramic material, such as HAp, or stiffness-enhancing polymers improves its performance for hard tissue engineering applications. The porous structure of chitosan provides space for bone cell growth. It can also be formed into porous structures used in cell transplantation or tissue regeneration. The most commonly used CaP phases in the fabrication of chitosan composites are HAp, TCP, and nanocrystalline or amorphous apatites [14,103]. Hydroxyapatite-chitosan-containing biocomposites are advanced, biocompatible materials widely used in bone tissue engineering, combining the structural support and osteoconductivity of HAp with the flexibility, biodegradability, and antibacterial properties of chitosan. These composites, often used as scaffolds or hydrogels, provide mechanically enhanced, porous environments that promote cell adhesion, proliferation, and differentiation, while reducing immune responses. Chitosan-hydroxyapatite biocomposites have been used in tissue engineering and in the treatment of inflammatory conditions, demonstrating favorable immunomodulatory properties. In these systems, chitosan generates a polymeric matrix and frames CaP particles. Biological tests showed [104] that the majority of bone marrow stromal cells adhered to the Ca-P/Ch layer after the first three days of culture, being predominantly bound to chitosan. Histological analysis results also showed that the addition of chitosan increased the probability of bone growth by up to 100%. Based on the synergistic effects of functionalization and doping, Mg/Sr-doped HAp/chitosan biocomposites have been developed for orthopedic applications [14,26,104,105,106,107]. They exhibited improved mechanical stability and controlled degradation, making them ideal for bone tissue engineering. Co-doping with Mg and Sr, combined with chitosan, improved biocompatibility and provided optimal morphological, crystalline, and structural properties for biomedical applications.

HAp-cellulose composites are biocompatible and biodegradable materials designed for bone tissue engineering, combining HAp, the osteoconductive mineral, with the structural and flexible framework of cellulose [108,109,110]. These scaffolds mimic natural bone architecture, promoting cell attachment, proliferation, and differentiation, while providing high mechanical strength for tissue regeneration.

Cellulose is the most widely available natural carbohydrate polymer. It is biocompatible, biodegradable, and hydrophilic. Biocomposites made with cellulose nanocrystals or bacterial cellulose are often used as a matrix for HAp, which acts as a filler. This improves biocompatibility and osteogenesis [104]. At the same time, the integration of HAp, especially in nanostructured form, significantly increases the compressive modulus and tensile strength of cellulose-based scaffolds, making them suitable for load-bearing bone applications. In addition to chitosan and cellulose, other natural polysaccharides (alginate, xanthan, fucoidan) have been used in combination with HAp to create composite biomaterials intended for tissue engineering applications, including those involving inflammation. In vitro studies on hydroxyapatite-alginate-chitosan (HAC) and hydroxyapatite-alginate-chitosan-fucoidan (HACF) hydrogels have indicated excellent biocompatibility and immunity [105]. These properties were considered to be due to the presence of natural polysaccharides, such as chitosan, which play an essential role in modulating immune responses.

#### 3.2.2. CaPs/Polymer Biocomposites

Collagen-hydroxyapatite composites contain biomaterials that mimic the natural organic (collagen) and inorganic (HAp) structure of bone. They combine high osteoconductivity, biocompatibility, and biodegradability, providing essential structural support for cell adhesion and proliferation. These biocomposites are widely used in the fabrication of scaffolds for the repair of bone defects. Collagen is a protein-based biopolymer and is among the main components of the ECM. This is the reason why they are used in the fabrication of porous scaffolds that mimic the ECM in supporting cell proliferation and organization. Studies conducted to date have proven the beneficial role of CaP/collagen composites for the repair of load-bearing bones. The influences of the porous structure of CaP, the collagen content, and the cross-linking method on the mechanical strength and biocompatibility of the scaffolds obtained in this way were investigated [111]. Since the use of collagen extracted from natural sources is limited because it causes immunogenic responses upon implantation, reconstructed collagen produced by biochemical processing has been used. In addition to the natural organization in the form of fibers, it has other polymeric formulations, such as gels, foams, and membranes. Since the use of collagen in scaffolds has the disadvantage of poor mechanical properties, collagen fibers have been cross-linked with polymers classified as natural or synthetic polymers [112,113,114,115].

Natural polysaccharides (chitosan, chitin, cellulose, starch) or proteins (gelatins) are used as natural polymers, and biodegradable synthetic polymers are poly(lactic acid), poly(glycolic acid), poly(lactic-coglycolic acid), poly(ε-caprolactone), and poly(lac-cocaprolactone). These polymers have been frequently used for scaffold fabrication due to their wide range of biodegradability. Also, these polymers can be used in the synthesis of copolymers with poly(ethylene glycol) (PEG) since the latter does not have the necessary biodegradability, but is a biocompatible hydrogel that has mechanical characteristics similar to certain soft tissues, such as cartilage [6,116,117,118].

Gelatin is a natural protein-based biopolymer with good biocompatibility. This polymer can be easily modified with different functional groups by coupling with crosslinking agents or other ligands. Gelatin-based composites are ideal candidates for tissue engineering. Thus, composites with HAp, gelatin, and transglutaminase as crosslinking agents have been prepared with a strength close to that of natural bones [119]. Proteins have been shown to regulate the size, particle size distribution, morphology, and assembly method of nanostructured CaPs, thus being used to fabricate novel materials with biomimetic characteristics for hard tissue repair [120].

Table 4 presents several biocomposites obtained by functionalizing CaPs using various methods. Their properties are multiple and prove their potential for use in tissue engineering.

#### 3.2.3. CaP Bioglass Composites

Among the bioactive composites of interest are those containing bioactive glasses [27,123]. Bioglasses (BGs) constitute the most extensively studied class of bioactive inorganic materials exhibiting osteogenic activity, and they have already progressed to commercial implementation [124]. Other functionalities of the bioglasses include magnetic or antibacterial properties, the ability to conduct electricity, and the release of oxygen [26]. Despite early development of the 45SiO_2_–24.5Na_2_O–24.5CaO–6P_2_O_5_ glass, named 45S5 Bioglass^®^, in 1969 by Hench, bioglass clinical uses are limited [124], due to inadequate mechanical properties, mismatching between the coefficient of thermal expansion (CTE) between BG’s coatings and supports, faster dissolution of the BG’s coatings, and crystallization tendency during high-temperature working processes, such as sintering and fiber drawing [125]. The first Bioglass^®^ devices were used in 1985 and 1988 to replace the middle ear bones and tooth bridges [125]. To enhance CaP performance and expand its range of applications toward soft and interface tissue repair as well as drug delivery, numerous alternative bioglass formulations have been developed.

Compositionally, BGs are silicate, silico-phosphate, and borosilicate glasses with different oxide additions. Analogous to CaP materials, the primary limitation of using bioactive glass (BG) to obtain porous scaffolds that mimic human bone texture is its inadequate mechanical properties, particularly brittleness [126,127]. The silica component in bioglasses significantly increases glass stability, compressive strength, and fracture toughness [127]. Few solutions are available for controlling BG crystallization, tailored composition, sol–gel preparation, understanding crystallization behavior, surface modifications, and polymer-BG composites [128]. The silica content, type of glass modifier (Na, K, Ca, Mg, and Ba [129]), and doping oxides (ZnO, Ce_2_O_3_, Ga_2_O_3_, Bi_2_O_3_, TiO_2_, and Nb_2_O_5_ [130,131,132,133], etc.) are critical factors determining the ability of BGs to crystallize [134,135] and obtain multifunctional BGs with antibacterial ability and angiogenic behavior. Although rich silica BGs up to 80 wt.% are still bioactive [136], they are denser than Hench’s Bioglass^®^ with 45% SiO_2_ [128]. Double P=O bonds in silico-phosphate glasses increase the crystallization tendency [136]. Generally, multiple crystallization effects have been reported in multicomponent bioglasses.

Improving the mechanical performance of a glass by controlled crystallization may compromise its bioactivity [127]. It is well known that crystallized glass ceramics have a less reactive surface in physiological solutions due to reduced surface Si-OH linkages compared to their glassy counterparts. Hence, brittleness and bioactivity are influenced by the crystallization behavior of BGs.

**Figure 4:**
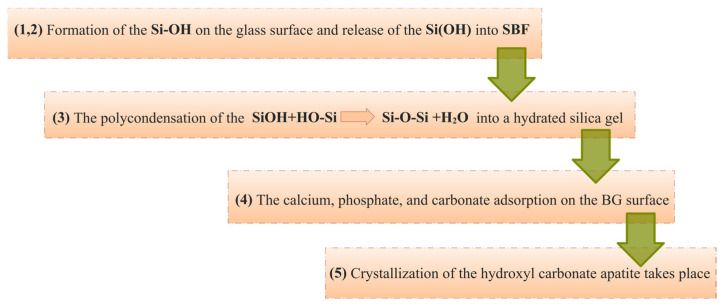
Sequential steps in the formation of the hydroxy-carbonate apatite (HCA) layer on BG surfaces.

Leaching, dissolution, and precipitation are the main processes for obtaining a hydroxy-carbonate apatite (HCA) layer on the surface of a BG when treated with simulated biological fluid (SBF) [137]. These processes take place during the first five stages of the 12 stages of bone formation [138]. The five stages described above (Figure 4) are controlled by the kinetics of the surface reactions [137].

**Figure 5:**
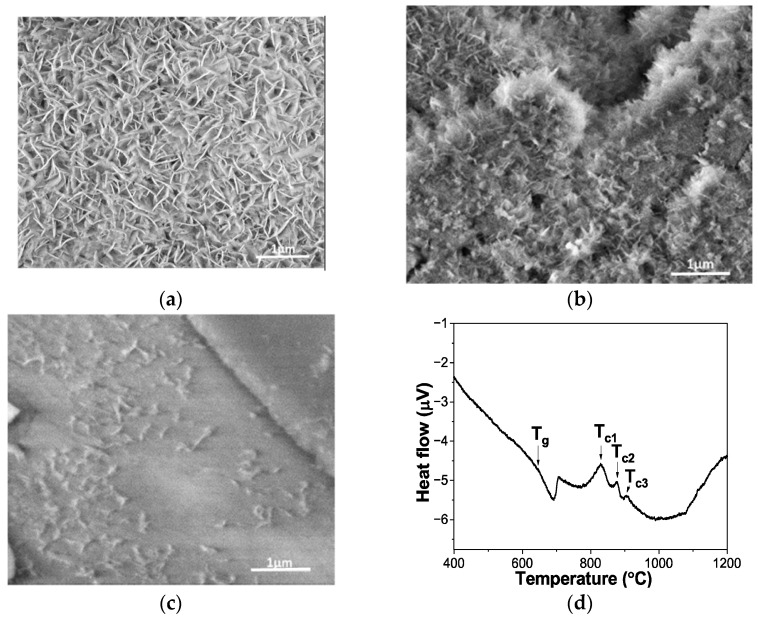
SEM micrographs of the 14-day immersed in SBF of: (**a**) bioglass, (**b**,**c**) isothermal crystallized bioglass-ceramics at the first two crystallization temperatures indicated by the DSC curve, and (**d**) DSC curve of the 70SiO_2_-21CaO-4P_2_O_5_-5CeO_2_ mesoporous bioglass (the third crystallization effect corresponds to ceria).

Glass formulation influences the duration of the mineralization stages. A ceria-doped mesoporous lime silicophosphate glass (70SiO_2_-21CaO-4P_2_O_5_-5CeO_2_) exhibited superior bioactivity in SBF after 14 days of immersion compared with its corresponding glass-ceramic derivatives (Figure 5a,b) obtained through isothermal treatments at the first two crystallization peaks corresponding to the apatite (Ca_5_(PO_4_)_2.823_(CO_3_)_0.22_O) and pseudowollastonite (CaSiO_3_) phases identified in the DSC profile in Figure 5d [139]. Hence, surface crystallization, probed by kinetic analysis of the DSC peaks of crystallization and UV-Raman spectroscopy [139], of this silica-rich bioglass caused a decrease in its bioactivity. Ceria-doped lime silicophosphate bioglasses mineralize faster than the CaP materials [140]. A reasonably large temperature window for processing (>100 °C) without crystallization was obtained for these CaO-SiO_2_-P_2_O_5_ mesoporous bioglasses doped with ceria in accordance with the difference between the onset temperature of the first crystallization peak and the glass transition temperature, Tg.

**Figure 6:**
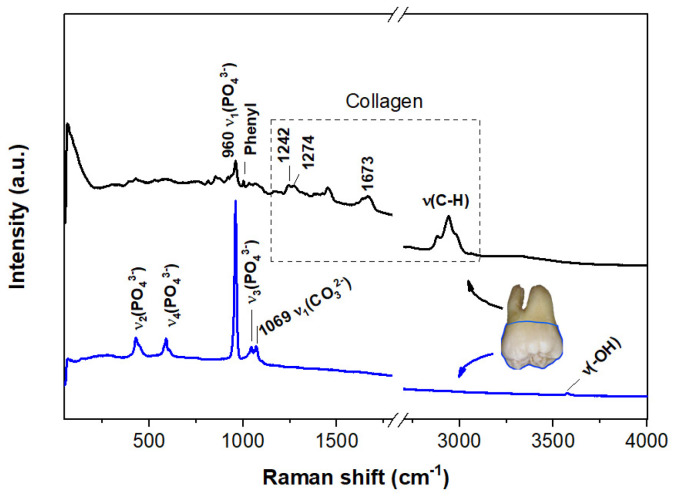
Raman spectra of tooth enamel and dentine [149].

Along with phase composition, the porosity and surface roughness of both bioglasses and CaP ceramics are essential for their osteointegration. Conversely, remineralization of the impaired tooth enamel, considered the most difficult HAp structure to replicate, requires porosity-free solutions [141]. Consequently, hydrogels formulated with natural polymers and polysaccharides have gained significant interest for promoting the remineralization of tooth enamel and dentine [142,143,144,145]. The latter hard tissue accommodates collagen in addition to a mineral phase consisting of phosphate, carbonate, and hydroxyl groups [146]. Among various spectroscopic techniques (Energy dispersive X-ray spectroscopy, EDS, coupled with SEM, photoelectron spectroscopy, XPS, FTIR spectroscopy, etc. [147,148]) for distinguishing between the dental material to be repaired and the remineralized one, Raman spectroscopy can identify mineral (phosphate, carbonate, and hydroxyl groups) and collagen phases of the enamel and dentine respectively [146,147]. Figure 6 illustrates visible Raman spectra of a natural demineralized molar slice [147]. A few Raman spectral features (peak positions, intensity ratios, and full width at half maximum, FWHM) were defined to differentiate between enamel and dentine before and after remineralization of hard tissues [146,149]. Tooth demineralization is depicted by the phosphate/carbonate area ratio, A(960)/A(1070). Large FWHM of the 960 cm^−1^ phosphate peak is an indication of the dentin or a remineralized tissue. Moreover, the Raman spectral collagen features of dentine are located within the 1200–1700 cm^−1^ range [146]. Mineral-to-collagen matrix ratio is derived from the 960/1455 peak area ratio. Replacement of the OH-groups of HAp by so-called A-type substitution in apatite materials, taking place in the remineralization processes, triggers Raman modifications in the 1000–1100 cm^−1^ range [150] as well as at about 3570 cm^−1^. Fluorescence is the main drawback of Raman investigations of remineralized tissues. However, fluorescence removal by UV-Raman spectrometers also enables accessing the molecular data at the surface of CaP materials [139].

In addition to melt quenching, sintering, the sol–gel method, and precipitation, the biomimetic method has been used for tailored HAp materials [142,143,144,151]. Biomimetic approaches to synthesizing nanocrystalline HAp often employ templates that replicate the collagen fiber network found in human bone, including polysaccharides, proteins, and various polymers [144,146]. During early tooth development, ameloblasts secrete proteins into the ECM that bind with calcium and phosphate ions, guiding the formation of highly oriented, parallel HAp crystallites in enamel. In biomimetic approaches, this natural gel-like ECM is therefore substituted with hydrogels to replicate its role in enamel formation.

Recently, it has been reported that bioglass-containing toothpaste can prolong tooth life by remineralization [152,153].

Mechanical limitations, processing scalability, and unknown in vivo behavior of bioglass-based systems in biomedical applications are challenging.

## 4. Biological Properties and Their Medical Applications

**Figure 7:**
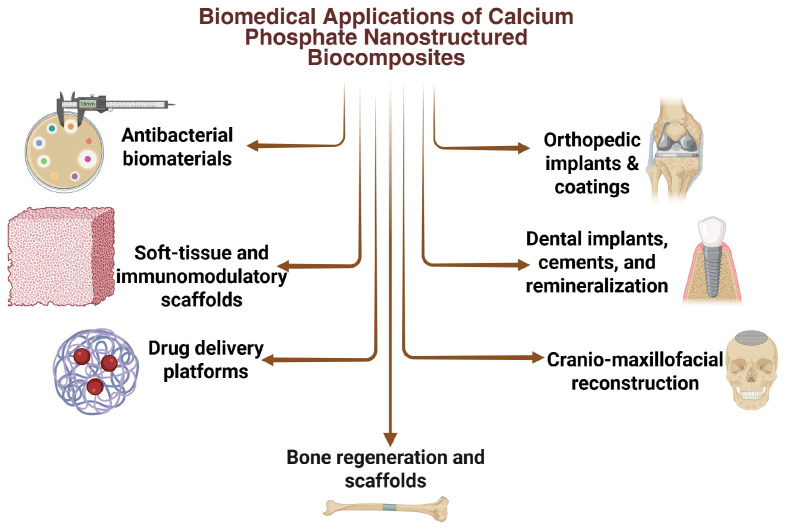
Schematic representation of major clinical application fields for CaP-based biocomposites. Created in BioRender. https://BioRender.com/6n9kve7, accessed on 1 January 2026.

Calcium phosphate-based nanostructured biocomposites exhibit additional biological properties that extend their usefulness beyond classical osteoconductivity, ion release, and antibacterial functions already described in previous sections and reflected in Figure 7. These emerging properties are essential for the development of multifunctional biomaterials capable of interacting with the physiological environment in more complex and therapeutically relevant ways.

### 4.1. Immunomodulatory and Pro-Healing Signaling Beyond Basic Biocompatibility

CaP bioceramics, long valued for their biocompatibility, are increasingly recognized as active regulators of immune responses rather than passive scaffold materials. Their surfaces rapidly adsorb proteins upon implantation, forming a protein corona that shapes macrophage activation, dendritic-cell maturation, cytokine release, and ultimately the foreign-body response. Native CaP materials often trigger excessive inflammation due to calcium-dependent signaling, but surface functionalization, through peptides, cytokines, antibodies, chemokines, or engineered topographies, enables precise immunomodulation. Functionalized CaP surfaces can promote anti-inflammatory M2 macrophage polarization, attenuate pro-inflammatory pathways, or, conversely, enhance immunogenicity for vaccine applications by presenting TLR ligands or antigens. These modifications transform CaP bioceramics into tunable immunomodulatory platforms capable of directing both innate and adaptive immune responses, although systematic studies of CaP—immune interactions remain limited and represent a critical area for future development [72].

Some works demonstrate that functionalized CaP materials can steer macrophage polarization, a critical determinant of tissue healing. Specific surface chemistries, nanoscale topographies, and biomolecule coatings (e.g., hyaluronic acid or cytokines such as IL-4 and IL-10) have been shown to promote anti-inflammatory M2 macrophage phenotypes, reducing the release of pro-inflammatory cytokines and supporting angiogenesis and osteogenesis at the implant site. Conversely, functionalization with pro-inflammatory cytokines (TNF-α, IFN-γ) or Toll-like receptor (TLR) agonists can intentionally enhance M1 polarization when an immune-stimulatory effect is therapeutically desirable, such as in vaccine delivery applications [72,154,155,156].

**Figure 8:**
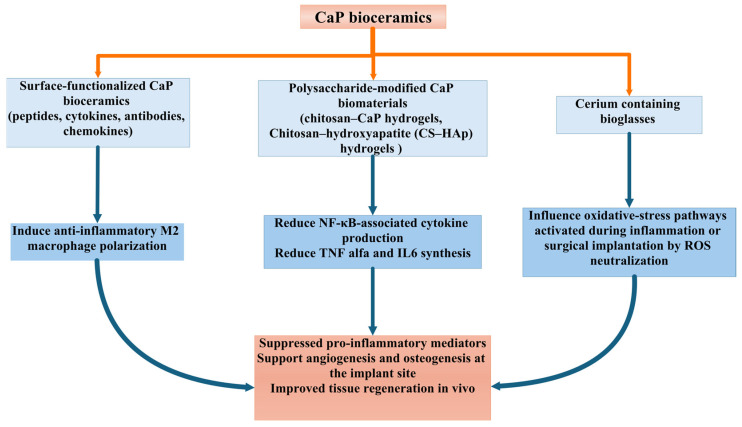
Calcium phosphate bioceramics and their functionalized derivatives express immunomodulatory mechanisms and support tissue regeneration.

Surface-functionalized CaP bioceramics also exert a significant influence over DC recruitment, antigen uptake, and maturation. By immobilizing specific ligands—including antibodies targeting DC-surface markers, TLR ligands (e.g., CpG DNA), or chemokines such as CXCL12—CaP NPs and scaffolds can modulate innate immune activation and shape downstream adaptive responses. CXCL12-engineered scaffolds can modulate innate immune activation by establishing a controlled, protease-responsive chemokine gradient that enhances early cellular infiltration and promotes coordinated wound healing [157]. This has led to the development of CaP-based platforms capable of inducing either strong immunogenic responses (e.g., for vaccines) or antigen-specific tolerance (e.g., for autoimmune therapies), depending on the nature of the conjugated biomolecules (Figure 8).

Another mechanism of immunomodulation is represented by the immune-adaptive surface chemistry. Polysaccharide-modified CaP surfaces (e.g., chitosan–CaP hydrogels) present functional groups that interact with immune cell receptors. These interactions dampen excessive inflammatory signaling and promote ECM deposition. Chitosan–hydroxyapatite (CS–HAp) hydrogels present chemical groups that interact with innate immune receptors on macrophages, influencing polarization and inflammatory signaling [105]. Chitosan’s protonated amine groups (–NH_3_^+^) electrostatically bind negatively charged macrophage membranes, enabling contact-dependent activation of macrophage pathways and affecting M1/M2 polarization. Chitosan modulates macrophage activation and neutrophil recruitment, indicating functional engagement of innate immune receptors, including pattern-recognition pathways [158]. The surface of HAp provides phosphate groups (PO_4_^3−^) and calcium ions (Ca^2+^) that promote the adsorption of serum proteins such as albumin, creating a protein corona that macrophages detect through integrins and scavenger receptors. This protein-mediated recognition dampens pro-inflammatory cytokine expression (TNF-α, IL-6) and supports a non-inflammatory macrophage response (Figure 8) [159]. In fucoidan-containing composites (HACF), the sulfated polysaccharide groups (−SO_4_^−^) can interact with macrophage lectin-like receptors and reduce NF-κB-associated cytokine production, further modulating innate immune signaling. Functional-group–driven receptor engagement collectively results in suppressed pro-inflammatory mediators and supports a shift toward a reparative macrophage phenotype, as reflected by reduced inflammatory cytokines and improved tissue regeneration in vivo [160,161].

Reduction in oxidative stress has been reported as the main antioxidant mechanism for composites containing cerium, manganese, or polysaccharide components. cerium-containing bioglasses modulate oxidative stress not by inducing ROS but by neutralizing them, largely through Ce^3+^/Ce^4+^ redox cycling that mimics endogenous antioxidant enzymes, promoting improved healing, reduced inflammation, and enhanced osteogenic responses [140]. Cerium-containing bioglasses (Ce-BGs) strongly influence oxidative-stress pathways because cerium cycles between Ce^3+^ and Ce^4+^ oxidation states. This redox cycling enables Ce-BGs to mimic antioxidant enzymes, but it also alters how reactive oxygen species (ROS) are generated or neutralized in biological environments. According to Nelson et al. [162], Ce-BGs do not induce oxidative stress; instead, they mitigate it by scavenging ROS and acting as catalase (CAT)- and superoxide dismutase (SOD)-mimetic agents [162]. When cerium is incorporated into bioglass, its redox-active surface rapidly engages ROS species produced during inflammation or surgical implantation (Figure 8). Excess ROS normally inhibits osteoblast differentiation and promotes osteoclast activity, exacerbating bone loss. Ce-BGs counteract this by converting ROS (such as H_2_O_2_ and superoxide) into harmless species. Their antioxidant behavior depends on the Ce^3+^/Ce^4+^ ratio, which is regulated by glass composition, phosphate content, particle size, and surface oxidation state. A higher Ce^3+^ fraction enhances SOD-mimetic dismutation of superoxide, whereas higher Ce^4+^ favors CAT-mimetic H_2_O_2_ degradation [163,164].

### 4.2. CaPs in Drug Delivery and Theranostics

Calcium phosphate cements function as adaptable local drug delivery platforms due to their bone-mineral-like composition, biocompatibility, injectability, and ability to harden in situ. CPCs have been used successfully to deliver antibiotics, anticancer agents, antiresorptive, anabolic molecules, growth factors, and even stem cells [165].

**Figure 9:**
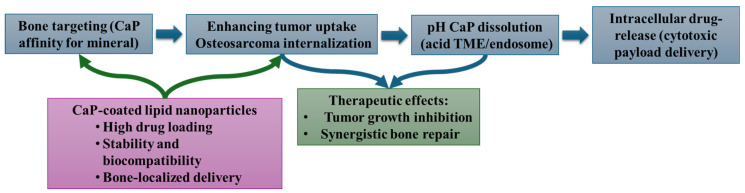
CaP nanostructures preferentially accumulate in bone tumors, enhance uptake by malignant cells, and dissolve in acidic tumor niches to release cytotoxic agents.

Antitumoral application. CaP nanostructures exhibit a natural affinity for bone mineral, enabling targeted accumulation in bone tumors. Their interfaces promote enhanced internalization by malignant bone cells such as osteosarcoma, followed by pH-triggered dissolution of CaP shells within acidic tumor microenvironments or intracellular vesicles. This dissolution triggers efficient intracellular release of cytotoxic payloads. CaP-coated lipid NPs further improve drug loading, biocompatibility, and bone-specific delivery, culminating in tumor growth inhibition and simultaneous support of bone repair (Figure 9).

CPCs have been successfully engineered to deliver a wide range of anticancer agents, including doxorubicin [166], cisplatin, etoposide, and 5-fluorouracil. Local chemotherapy via CPCs provides high intralesional concentrations capable of inhibiting residual tumor cells and reducing recurrence following bone tumor resection, while limiting systemic exposure. Incorporation of magnetic particles or therapeutic radioisotopes further extends CPCs into theragnostic systems, enabling localized hyperthermia or radiotherapy within the bone defect [167]. In addition, ion substitutions such as strontium, magnesium, or zinc can enhance osteogenesis and contribute to improved reconstruction of tumor-induced bone defects while maintaining compatibility with embedded antineoplastic agents [168].

Advances in scaffold engineering—such as macropore formation, polymer reinforcement, nanocarrier incorporation, and 3D printing—allow the fabrication of CPCs with optimized release kinetics, improved mechanical stability, and personalized geometries for segmental tumor reconstructions. These multifunctional CPC systems act simultaneously as structural fillers and localized drug depots, enabling coordinated bone regeneration and antitumor therapy. As biodegradable, customizable, and multifunctional matrices, CPC-based platforms represent a promising strategy for localized management of primary and metastatic bone tumors within modern theragnostic frameworks [53].

In 2024, Weng and collaborators published a study regarding the low tissue accumulation and off-target toxicity of systemic delivery, signaling a broader future for CaP NPs in non-invasive cardiovascular therapy [169]. This study introduces an inhalable cardiac-targeting CaP NPs system (TP-10@CaP-CTP) designed to prevent pressure-overload–induced heart failure. Inhalation enables rapid lung-to-heart transport through the cardiopulmonary circulation, producing greater and faster myocardial accumulation than intravenous delivery. CTP selectively targets pathological, but not healthy, myocardium—binding diseased cardiomyocytes (CMs) and cardiac fibroblasts (CFs) while avoiding lung cells—thus improving cardiac specificity at low drug doses. Mechanistically, the released PDE10A inhibitor TP-10 restores intracellular cyclic nucleotide signaling. In cardiomyocytes, TP-10@CaP-CTP elevates cAMP, activating AMPK, which reverses hypertrophic growth and suppresses markers such as Nppa, Nppb, and β-MHC. Blocking AMPK eliminates these benefits, confirming a cAMP→AMPK cardioprotective pathway. In cardiac fibroblasts, the NPs increase cGMP, activating PKG, which inhibits proliferation, migration, myofibroblast transition, and ECM synthesis.

**Figure 10:**
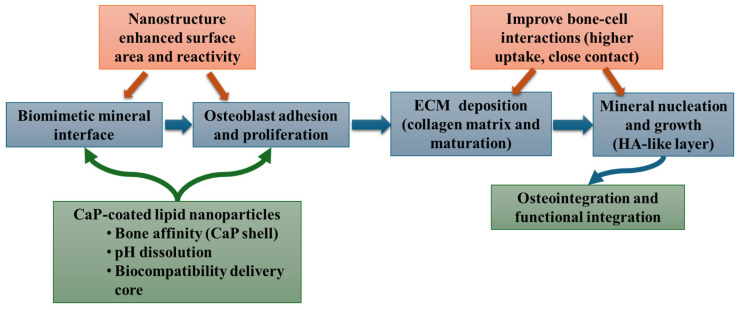
CaP-assisted bone regeneration mechanism. CaP interfaces mimic the mineral phase of bone (biomimicry), promote osteoblast adhesion and proliferation, support ECM deposition, and drive HAp-like mineral nucleation, culminating in osseointegration. Nanoscale CaP increases surface reactivity and cell–material interactions; CaP-coated lipid NPs further enhance cell uptake and bone affinity, enabling targeted delivery at regeneration sites.

From a biological standpoint, CaP-coated NPs demonstrated significantly enhanced cellular uptake in human osteosarcoma cells, compared to uncoated lipid NPs. While the study used osteosarcoma cells as an in vitro model, the results remain relevant for bone regeneration research: improved internalization indicates better interaction between NPs and bone-related cells, which is a prerequisite for delivering osteogenic cues or bioactive molecules during regeneration processes. Furthermore, all CaP-coated formulations exhibited excellent biocompatibility, showing no cytotoxic effects even at high concentrations. Non-toxic behavior is essential for bone-regenerative biomaterials, as osteoblast viability is critical for effective bone matrix deposition and mineralization. The ability of CaP-coated NPs to deliver a hydrophobic model compound more efficiently to cells indicates their potential to transport osteoinductive agents such as growth factors, differentiation inducers, or anti-inflammatory molecules directly to bone-forming cells. This targeted intracellular delivery could support osteogenic differentiation and enhance bone regeneration outcomes (Figure 10) [170].

### 4.3. Antimicrobial Properties and Mechanisms of CaP Materials

**Figure 11:**
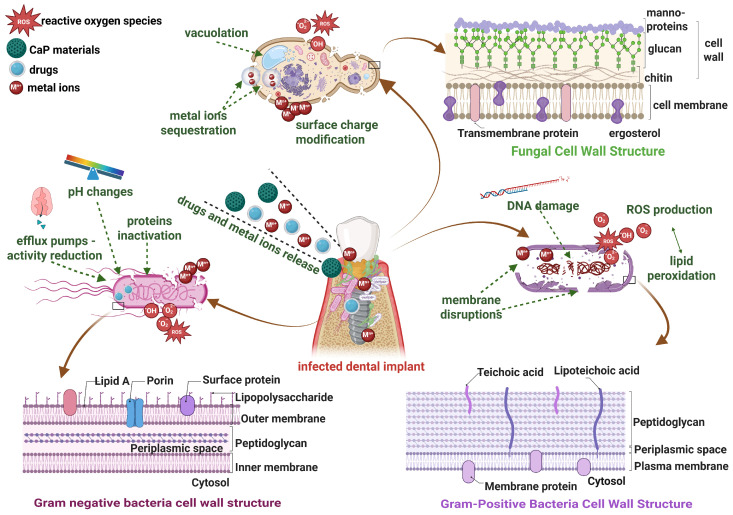
The principal antimicrobial mechanisms of CaP-based compounds. Created in BioRender. Ditu, L.-M. (2026) https://BioRender.com/wpb78xe, accessed on 1 January 2026.

Currently, bacterial and biofilm-associated antimicrobial resistance remain significant challenges for researchers, and the development of biomaterials must contain several properties according to their scope, particularly to design principles, antimicrobial surfaces, and their mechanisms of action [171]. Moreover, the new generation requires antimicrobial properties of the materials and multifunctionality, independent of the traditional treatment with antibiotics [172]. Following, the main antimicrobial mechanisms of CaP-based nanocomposites (Figure 11), such as generation of reactive oxygen species (ROS), disruption of microbial cell membrane, interference with bacterial adhesion and early biofilm formation, generation of local pH shifts, chelation and nutrient sequestration effects, and electrostatic and chemical interference with bacterial cell wall components, will be discussed in detail.

Antibacterial nanostructured surfaces developed by biomineralization of calcium phosphate precursors significantly inhibited both *P. aeruginosa* and *S. aureus* antibiotic-resistant bacteria up to 75% in adhered bacteria, only after 4 h of incubation [173]. The CaP-based nanoneedles induced simultaneously *oxidative stress/high ROS levels* and *membrane damages* (Figure 11). Otherwise, the significant mechano-bactericidal properties do not alter the bioactivity of the materials, which can serve as antibacterial and osteogenic grafts [173]. It is known that calcium is involved in microorganisms’ integrity and metabolism, but higher extracellular calcium levels affect the bacterial envelope and significantly *inhibit the development*, *adherence*, *and structure of biofilms*, respectively. Moreover, the high levels of calcium negatively influence the fungus or viral infections [174]. In another study, Wu et al. [175] reported the fundamental theories behind the disruption of bacterial membrane mechanisms of amorphous CP and HAp. Firstly, the higher surface reactivity of amorphous CP plays a key role in inhibitory effects against *E. coli* and determines a more pronounced membrane damage than HAp. Also, changes were observed in the lipopolysaccharides structures of the *P. aeruginosa* membrane. Likewise, the bacteriostatic effects against *S. aureus* and damages of cell membrane were observed in modifications of a lipoteichoic acid, especially of the C=O ester carbonyl stretch. Otherwise, HAp produced damage to the methicillin-resistant *S. aureus* membrane by *reducing the activity of overexpressed efflux pumps*. As well, the disruption of bacterial membranes was reported by Ganbaatar et al. [176]. The polycaprolactone- calcium phosphate composite exhibited mechano-bactericidal effects against *E. coli* and *B. subtilis*, and promoted the adhesion, proliferation, and differentiation of pre-osteoblasts. The SEM images indicated that *E. coli* membrane morphology was more affected by the developed composite than *B. subtilis* membrane, probably due to the limited resistance of the lipopolysaccharides layer of the Gram-negative bacteria membrane.

The *doping composites* with different *metal ions* enhance the antimicrobial properties due to the *multiple pathways of inhibitory effects* (Table 5, Figure 11), such as ion release, membrane disruption, oxidative stress, etc. [177]. According to the chemical and physical properties of metal ions, they can influence different factors, such as pH, temperature, ROS generation, ionic strength, etc., and can interfere with bacterial cell components, enzymes, or DNA [178]. Moreover, the metal ions significantly influence bioavailability and microbial properties of materials due to a complex network of transporters, metalloregulator sensors, and chelation—metal storage processes [179,180].

The metal ions were attached to the bacterial membrane by electrostatic, receptor-ligand, or hydrophobic interactions, and van Der Waals forces. Depending on their shapes, the metal ions traverse the bacterial membrane and generate oxidative stress, inhibiting enzymes, deactivating proteins, changing the gene expression levels, and, in other words, inducing metal toxicity [201].

Initially, the metal ions and metallic NPs interact with the bacterial cell envelope, which is the most important barrier between the external environment and the cell. Moreover, the bacterial cell envelope ensures the electron transport chain, and external metal ions can be imbalanced in it, changing the local pH, respectively [177,202]. Additionally, the bacterial structure consists of sites of enzymes and proteins, like metal-dependent β-lactamases, metallochaperones, metalloenzymes, etc., that can sequester the metal ions [203]. An important component is represented by lipids, especially phospholipids, which, after interaction with ions, are imbalanced by chain processes, such as polarity, local pH, decrease in dipole potential, imbalance of cellular function, and of the surface charge, generating ROS formation, and appearance of membrane disruptions. Secondly, the high amounts of metal ions in the membrane lead to damaging the cell barrier and leakage of the intracellular content [204]. In general, Gram-positive bacterial strains are sensitive to the action of zinc, copper, samarium, and gadolinium, while silver and selenite determine inhibitory effects on Gram-negative bacteria [205].

Furthermore, the main characteristics of metal- induced ROS production are: (i) participation of metal ions in reduction/oxidation processes by increasing or losing electrons, (ii) disruptions of the cellular donor ligand that coordinates Fe and promote an uncontrolled release into cytoplasm, where they generate ROS, and (iii) triggering oxidative stress by diminishing the antioxidants’ deposits [206,207,208].

The antifungal mechanisms of metal ions consist of the generation of ROS, interaction and damage of the cell wall, hyphae, and spores, deformation of the plasma membrane, inhibition of spore germination, disruption of gene and protein regulation, and alteration of biofilm architecture (Figure 11) [209,210].

The fungal cell wall plays a fundamental role in growth, defense, morphogenesis, and biofilm development. It serves as a protective interface that mitigates osmotic pressure fluctuations, detects environmental cues, and shields the organism from adverse conditions such as desiccation, higher temperatures, and exposure to toxic compounds, like unessential metal ions. In pathogenic fungal species, the cell wall is additionally essential for virulence, facilitating host invasion while providing protection against immune defense mechanisms. The cell wall is composed primarily of polysaccharides and proteins, including chitin, glucans, mannans, glucanases, and various glycoproteins. Even if it presents a rigid structure, the cell wall is highly dynamic, undergoing continuous remodeling in response to cellular proliferation processes—such as expansion, sporulation, and hyphal branching—and to changing environmental conditions. Cellular events like binary fission or hyphal extension require coordinated anabolic and catabolic enzymatic activities to restructure the wall in an integrated and suitable way [211].

In yeast, metal resistance primarily appears from the downregulation of metal ion import systems, the deployment of metallothioneins and metallothionein-like molecules, and the sequestration of excess ions within the vacuole (Figure 11). By contrast, filamentous fungi rely predominantly on the active export of metal ions to confer resistance. Nonetheless, additional resistance mechanisms have been documented in filamentous species, including vacuolar sequestration, binding of ions by metallothioneins and other chelators, deletion or suppression of metal ion importers, and the storage of metal ions within the hyphal cell wall matrix. Overall, resistance mechanisms for zinc, copper, iron, and manganese are well-characterized in yeast, whereas the corresponding processes in filamentous fungi remain only partially understood [212]. However, metal ions in excess or toxic, like silver, induce modifications in the hyphae, which change into twisted, shrunken, and shriveled. Moreover, the abnormal mycelial morphology is linked to increased permeability of the mycelial cell membrane, noticeable vacuolation, and loosening of structure. Besides mycelium and organelle degradation, the silver ions are also responsible for changes in carbohydrate metabolism, the TCA cycle, and energy metabolism pathways (Figure 11) [213].

On the other hand, the antimicrobial properties of the CaP materials can be enhanced by using them as a carrier for drugs by combining/loading with antibiotics (Figure 11) [214]. Moreover, amorphous CaP and HAp exhibited synergistic effects with several antibiotics, including ampicillin, kanamycin, oxacillin, vancomycin, minocycline, erythromycin, linezolid, and clindamycin, against standard and clinical strains, like *S. aureus*, *S. epidermidis*, *E. faecalis*, *E. coli*, and *P. aeruginosa*. The materials displayed a complex relationship with drugs, in which the inhibitory effects are proportional to the CaP and HAp quantities, and generated a concentration-dependent minimum to stress-induced biofilm development [215].

## 5. Challenges and Perspectives

Additive manufacturing, stimuli-responsive CaP systems, and personalized biomaterials are particularly promising for the next generation of personalized medical devices, such as bone regeneration/substitution or dental substitution materials [216,217].

Additive manufacturing refers to 3D printing, which enables the creation of scaffolds and implants that mimic the natural architecture of bone, customized for every patient [218]. Moreover, additive manufacturing enhances the biocompatibility and efficiency of materials such as ceramics, alloys, composites, etc., and presents lower risks. The foremost additive techniques consist of stereolithography, digital light processing (DLP), fused deposition modeling, selective laser sintering, selective laser melting, and syringe-based extrusion [219,220]. For example, in a recent study, Wei et al. [221], reported an optimized formula for the fabrication of osteoinductive CaP ceramics through the DLP method. The authors underlined the importance of high precision in the manufacture of customized CaP materials for bone-repairing products. In another research, Duque et al. [222] obtained biocompatible polymeric scaffolds from a commercial resin loaded with CaP NPs through the DLP approach. Correspondingly, de Carvalho et al. [223] used the extrusion technique for fabricating 3D-printed scaffolds based on mesoporous BG with applicability in bone tissue engineering. Similarly, Bertol et al. [224] applied 3D-printing extrusion and computer-aided design (CAD) approaches for developing accuracy and patient-customizable craniofacial implants, which are based on α-TCP and Na_2_HPO_4_. Also, the extrusion technique can be combined with controlled deposition for the fabrication of β-TCP and HAp-based scaffolds with interconnected macroporous architecture for bone repair applications [225].

On the other hand, the additive manufactured products have relatively higher prices due to the complexity of the entire processes, such as scanning analyses of patients, personalized digital design, software, complex equipment and maintenance, experienced researchers and operators, costs of printing, maintenance, biomechanical simulation, and surgical operation for implantation [226]. Currently, geometrical data obtained from CAD software and magnetic resonance imaging (MRI) scans are used for the manufacturing of patient-specific CaP osteochondral implants through 3D-printing extrusion processes [227]. Moreover, the authors highlighted the importance of an interdisciplinary team (engineers, radiologists, and surgeons) and the strategies for minor intraoperative adjustments of the implants [227]. Likewise, Anderson et al. [228] underlined the significance of cone beam computed tomographic imaging and 3D printing for the fabrication of customized clinical-scale and patient-specific bioceramic scaffolds. The Osteoink^TM^ scaffolds with high compressive strength were based on CaP bioceramics (alginate/β-TCP and HAp/α-TCP). Otherwise, despite some limitations of additive manufacturing, such as shrinkage of ceramics, tool resolution, requirement of a supportive material, etc., the formative technology can be an alternative method [229]. Parfenov et al. [229] fabricated 3D scaffolds using magnetic levitation of CaP particles, and the rapid chemical transformation of TCP into OCP enhances the biocompatibility of the material.

Multifunctional design and CaP material-biological pairing improve the biological performance of developed materials by targeting osteo-immunomodulatory regulation, biochemical activation, and ECM-mimetic functionalization [230]. Additionally, biomimetic strategies include the integration of CaP with polymeric or bioactive inks to simulate the native bone microenvironment, leading to enhanced osteointegration and modulation of biological responses. For instance, Torres et al. [231] developed CaP-based 3D-printed scaffolds with enhanced bioactivity due to the incorporation of human platelet lysates, which are a valuable source of growth factors, cytokines, and chemokines. In addition, the human platelet lysates increase the bioactivity of materials, and the scaffold’s macroporous design assists bone ingrowth and integration. Also, the developed 3D-printed scaffolds allow personalization and offer a clinical solution for bone repair and customized regenerative therapies. Furthermore, the integration of artificial intelligence, like AI-guided design, can accelerate the development of prostheses and will have a substantial impact on personalized medicine [232,233].

Stimuli-responsive CaP systems enhance the adaptability and biological response of the newly designed implants. Integration of pH-sensitive, antioxidant or infection-responsive molecules can reduce or remove the inflammatory processes associated with directly targeting the immune system [234,235]. Thus, cytochrome C is used as a pH-sensitive molecule for a better pH-dependent release control and stimuli responsive of a 3D-printed silica platform/scaffold [236]. A recent research [237] reported the significance of the antibiotic loading in 3D-printed CaP scaffolds on *S. aureus* infection management of rabbits. Gentamicin, rifampicin, cefazolin, and vancomycin were used, and the study demonstrated that a single type of antibiotic loaded on implants is insufficient to eradicate the infection and requires supplementation with systemic therapy. On the other hand, depending on the type and anatomic functionality required for the implants, a high interest is represented by the development of shape memory and dynamic materials due to a better structural adaptation, stimuli-response, and bone reconstruction [238].

Manufacturing materials that closely mimic natural bone is a big challenge, requiring multi-disciplinary approaches and a correlation between advances in manufacturing techniques, developments in medicine (especially in immunomodulation), and the design of personalized devices in accordance with international guidelines, such as Good Manufacturing Practice, Food and Drug Administration, European Medicines Agency, etc. [239].

## 6. Conclusions

Nanostructured calcium phosphate (CaP) biocomposites represent a highly versatile and bioactive class of materials for bone tissue engineering. Their chemical similarity to native bone mineral, combined with tunable morphology, crystallinity, and ion-release profiles, enables strong osteoconductive and regenerative performance. Functionalization through ion substitution, polymer integration, bioglass incorporation, or biomolecule immobilization further enhances mechanical strength, bioactivity, immunomodulation, and antimicrobial effects.

The combination of CaP phases with natural or synthetic polymers produces hybrid scaffolds that closely mimic the ECM and support cellular adhesion, proliferation, and differentiation. Meanwhile, metal-ion doping offers effective antibacterial and antifungal properties, addressing implant-associated infections—a critical challenge in orthopedic applications. Additionally, CaP-based carriers show promising capabilities for targeted drug delivery and theragnostic, particularly in localized cancer therapy and personalized regenerative approaches.

Overall, the rapid progress in CaP nanostructuring and composite design demonstrates significant potential for next-generation biomedical implants. Continued efforts should focus on optimizing structure–function relationships, understanding immune interactions, and advancing clinically translatable, multifunctional biomaterials for improved bone repair and infection control. Continued integration of materials science, nanotechnology, biology, and clinical research will be critical for translating these innovations into effective therapeutic solutions.

## Figures and Tables

**Table 1:** Relevant publications on calcium phosphate composite materials.

The Main Discussed Subjects	Applications	Ref.
Bioactive calcium phosphates, bioactive chemical composition, synthesis methods, nanostructure, pore sizes, morphology, and mechanical performance	Regeneration of bone tissue	[24]
Calcium phosphate bioceramics, history, structure, CaP coating, clinical and industrial applications, development, perspectives	Bone regeneration, orthopedics and dentistry)	[25]
Calcium phosphate-containing biopolymers, orthopedic applications, recent progress, mechanical, chemical, and biological performances	Bone substitutes (implant, coating)	[26]
Bioactive glass (BG), glass -ceramics, osseointegration, osteoconductivity	Bone regeneration	[27]
Calcium phosphate biomaterials, biological properties including osteoinductivity, osteoconductivity, and biodegradability, clinical requirements, biodegradability, surface modifications, limited mechanical strength	Bone repair	[28]
Calcium phosphate-based biomaterials, bioactivity and better integration with host tissues, functionalized with metal ions, bioactive molecules/protein, multifunction strategies	Bone regeneration	[29]
Nanomaterials based on HAp, technology and computational techniques, morphological, mechanical, and biological properties, tissue regeneration	Tissue regeneration	[30]
Tissue regeneration, bioactive and osteoconductive properties, cement systems, functionally graded materials, future opportunities, biomimetic process and functionality of materials	Dentistry	[31]
Calcium phosphate-based biomaterials, physicochemical properties, ceramic or composite scaffolds with polymers, hierarchical nano-/microstructures, interactions between the physicochemical/biological properties of CaP biomaterials	Bone defect repair	[32]

**Table 2:** Correlation between structure, properties, and biomedical functionality of CaP materials.

CaP Type	Formula/Structure	Key Properties	Biomedical Functionality/Applications	Refs.
DCPD	CaHPO_4_·2H_2_O (brushite); CaHPO_4_ (monetite)	Low Ca/P ratio (1.0); high solubility; rapid degradability; fast resorption; metastable phase	Fast-setting CPCs *; dental cements and restorative materials; promotes extensive bone remodeling and ion release	[38]
TCP	Ca_3_(PO_4_)_2_; α-TCP, α′-TCP, β-TCP	Excellent biocompatibility; osteoconductive; polymorph-dependent solubility and stability; moderate to high resorbability	Bone graft substitutes; temporary scaffolds; α-TCP in CPCs; β-TCP as granules and blocks	[39,40]
TTCP	Ca_4_(PO_4_)_2_O	High reactivity; alkaline nature; cement-forming capability	Injectable/moldable CPCs; in situ hardening bone fillers	[36]
OCP	Ca_8_H_2_(PO_4_)_6_·5H_2_O	Metastable at physiological pH; osteoconductive; precursor to HAp	Bone substitute; nucleation site for bone deposition; enhanced bone formation	[41]
ACP	Non-crystalline CaP	Highly reactive; low stability; transient precursor; rapid conversion to HAp	Bone mineralization precursor; CPC modifier; drug delivery potential	[41,42]
HAp	Ca_10_(PO_4_)_6_(OH)_2_;	High biocompatibility; osteoconductive; slow degradation;	Bone and dental reconstruction; coatings; scaffolds; ALP/BMP ** stimulation	[38,43]

* CPCs = calcium phosphate cements; ** ALP/BMP = alkaline phosphatase/bone morphogenetic protein.

**Table 4:** Overview of CaP-based nanocomposites with biomedical applications, synthesis methods, and properties.

Nanocomposites	Preparation Method	Properties	Applications	Ref.
*n*Mg/Sr-HAp@CS (*n* = 0, 0.2, 0.4, 0.6)	HAp co-doped with Mg/Sr incorporated into chitosan matrix	high resistance to degradation through biological media	implant materials	[14]
Celulose/HAp	chemical and mimic of natural process of HAp growth on the surface of CNW, physical NPs suspension, and aggregation	improved mechanical properties, maintaining biological activities	bone tissue engineering	[102]
Collagen/HAp	synchronous mineralization and fibril reassembly	improving the mechanical properties as porous scaffold	implant material	[18]
Chitosan/TTCP phosphate and α-TCP	injection of a mixture	osteogenic and osteoconductive properties	implant material	[106]
Polylactic acid/TCP composites	fused deposition modeling	osteoblastic-like cell performance	scaffolds for tissue engineering	[115]
Calcium phosphate—chitosan	electrochemical deposition	osteogenesis properties	tissue engineering	[104]
Chitosan/agarose/HAp Chitosan/β-1,3-glucan (curdlan)/HAp	sintering	biomedical potential, bioactivity, bioabsorbability	tissue implantation, orthopedics, and maxillofacial surgeries	[121]
Sericin/CaPcomposites	wet mechanochemical processes (biomineralization)	bioactivity	biomedical engineering	[122]
Bacterial cellulose, HAp, graphene oxide	in situ and ex situ ultrasonication	bioactivity	bone tissue engineering	[109]
HAp-CaO-collagen	mechanochemical processes	slower degradation	bone graft	[26]

**Table 5:** Metals doped CaP-based materials with antimicrobial properties.

Type of Materials	Metal Ions	Microbial Strains	Antimicrobial Mechanisms	Biomedical Applications	Ref.
Ag^+^-BG-ceramic particles	Ag^+^	methicillin-resistant *S. aureus*	oxidative stress (ROS production), osmotic effects, bacterial membrane disruptions	orthopedic	[181]
HAp dopped	Ag^+^, Cu^2+^, Zn^2+^	*E. coli*, *S. aureus*	disruption of cell walls and membrane, generation of ROS, inhibiting enzymatic activity, protein and DNA denaturation	infection prevention, implants	[182]
Ag/Ca-P coating	Ag^+^	*S. aureus*	bacteria reduction via contact killing due to the leaching of formed AgCl_x_	medical implants	[183]
Chitosan/Ag@HAp	Ag^+^	*E. coli*, *S. aureus*	bacterial adhesion disruption, damage to the bacterial cell membrane, oxidative stress, and ROS production	multifunctional material	[184]
quaternary ammonium dimethacrylate-silver and amorphous CaP	Ag^+^	*Streptococcus mutans*	reduced the activity and acid production of *Streptococcus mutans*	dentistry (dental caries inhibition)	[185]
Ag-doped Ca_3_(PO_4_)_2_/MoS_2_ nanocomposites	Ag^+^	*S. aureus*, *S. epidermidis*, *E. coli*, *P. aeruginosa*	continuous release of Ag^+^ ions, pH changes, and direct interaction with bacteria due to the hydrophilicity of the composite	biomedical devices	[186]
Strontium-doped HAp	Sr^2+^	*S. aureus*, *L. monocytogenes*, *S. enteritidis*, *A. baumanii*	positive surface charge and direct interaction with the bacterial cell membrane	implants and scaffolds	[187]
Gelatin-BG-strontium	Sr^2+^	*E. coli*, *S. aureus*	pH changes	bone regeneration, tissue engineering	[188]
Sr-TCP coating	Sr^2+^	*E. coli*, *S. aureus*	inhibited bacterial adhesion, reduced biofilm formation, and altered biofilm architecture	orthopedics	[189]
Sr^2+^, Cu^2+^ phosphate glasses	Sr^2+^, Cu^2+^	*S. aureus*	ion exchange release, pH changes	healthcare and public infrastructure	[190]
Strontium-copper-substituted phosphates: Ca_9.5−x_Sr_x_Cu(PO_4_)_7_	Sr^2+^, Cu^2+^	*E. coli*, *S. aureus*	synergistic effect of co-doping, high release of metal ions	bone implants	[191]
Multifunctional CaP doped with strontium and zinc	Sr^2+^, Zn^2+^	*E. coli*, *S. aureus*	The release of Sr^2+^ and Zn^2+^ inhibited the biofilm formation and promoted the implant osseointegration	orthopedic implant, biomimetic apatite mineral	[192]
Zinc-doped SrCaP on a titanium implant	Zn^2^	*E. coli*, *S. aureus*	inhibited and damaged the biofilm structures	implants	[193]
Co-doping ZnO and Zn^2+^ in CaP ceramics	Zn^2+^	*E. coli*, *S. aureus*	ROS generation, protein inactivation, ZnO deposition, and synergistic antibacterial effects	regenerative repair	[194]
Metal oxide-doped CaP glasses	Zn^2+^, Co^2+^, Cu^2+^	*S. aureus*, *E. coli*, *C. albicans*	penetration of metal ions through the outer membrane, cationic sequestering	antimicrobial bioactive glasses	[195]
Copper-HAp functionalized multiwalled carbon nanotube	Cu^2+^	*B. subtilis*, *S. aureus*, *S. flexneri*, and *E. coli*	Spherical Cu^2+^ ions generate ROS accumulation, plasmid DNA damage, breakdown the permeability barrier, membrane disruptions, and a bacterial apoptosis-like response	implants	[196]
Magnesium-doped HAp	Mg^2+^	*P. aeruginosa*	biofilm inhibition due to the Mg^2+^ presence in the lattice of the HAp	antimicrobial coatings	[197]
Manganese-doped BG	Mn^2+^	*E. coli*	pH modification of media, association of Mn^2+^ with thiol groups	bone implants	[198]
Ce^4+^/Si^4+^ doped HAp	Ce^4+^/Si^4+^	*S. aureus*, *B. subtilis*, *E. coli*, *P. aeruginosa*	electrostatic interaction with the bacterial cell wall, binds to the thiol group of the protease enzyme, inhibiting the signaling activity, enzymatic, and cellular respiratory processes	Multifunctional coating-biomaterial for bone regeneration	[199]
Cerium-HAp and β-TCP scaffolds	Ce^4+^	*S. aureus*	surface charge inhibited the *S. aureus* growth	orthopedics	[200]

## Data Availability

No new data were created or analyzed in this study. Data sharing is not applicable to this article.
